# Development and Characterization of Printlets of Lamivudine for Pediatric Patients Using Selective Laser Sintering

**DOI:** 10.3390/ph18030356

**Published:** 2025-03-01

**Authors:** Canberk Kayalar, Swaroop Jalandar Pansare, Gereziher Sibhat, Mathew Kuttolamadom, Ziyaur Rahman, Mansoor A. Khan

**Affiliations:** 1 Irma Lerma Rangel College of Pharmacy, Texas A&M Health Science Center, Texas A&M University, College Station, TX 77843, USA; kayalarc@tamu.edu (C.K.); swaroop0205@tamu.edu (S.J.P.); sibhat_23@exchange.tamu.edu (G.S.); rahman@tamu.edu (Z.R.); 2 The Department of Engineering Technology and Industrial Distribution, Texas A&M University, College Station, TX 77843, USA; mathew@tamu.edu

**Keywords:** lamivudine, selective laser sintering, printlets, dissolution, pharmacokinetics

## Abstract

**Background:** Lamivudine is widely used alone or in combination with other anti-HIV drugs in the infant to adolescent age groups of pediatric populations. Compounding of medications is frequently used for pediatric patients. However, many issues have been reported for the compounded formulation such as assay, stability, safety, and efficacy. Three-dimensional printing can overcome these issues. **Objective:** The aim of this study was to understand the effect of process and formulation variables on lamivudine printlets for pediatric populations using selective laser sintering. **Methods:** The Plackett–Burman screening design was used to prepare 12 formulations to study six variables, namely, laser scanning speed (130–150 °C), surface temperature (105–120 °C), chamber temperature (250–350 mm/s), sucrose (0–30%), hydroxypropyl methylcellulose (0–42%), and Kollidon^®^ CL-M (0–5%). The formulations were tested for dissolution, disintegration, hardness, assay, X-ray diffraction, differential scanning calorimetry, stability, and pharmacokinetics in Sprague Dawley rats. **Results:** The assay of the printlet formulations varied between 93.1 and 103.5% and the disintegration time was 2.8 ± 1.2 (F1) to 43.7 ± 2.7 (F10) s. Due to high surface temperatures, the unsintered powder in the printing chamber experienced significant changes in crystallinity. No statistical significance was observed between the pharmacokinetic parameters of the printlets and commercial tablets (*p* > 0.05). The maximum plasma concentration (C_max_), time to reach maximum plasma concentration (T_max_), and area under the curve (AUC) of the printlets and commercial tablets were 295.5 ± 33.0 and 305.0 ± 70.1 ng/mL, 0.5 ± 0.0 and 1.0 ± 0.8 h, and 1414.1 ± 174.0 and 1987.2 ± 700.5 ng.h/mL, respectively. **Conclusions:** In summary, fast-disintegrating and dissolving 3D printed lamivudine was found to be bioequivalent to commercial formulation of lamivudine. Thus, it is a viable method for dispensing personalized lamivudine printlets for pediatric populations.

## 1. Introduction

Lamivudine (3TC), a nucleoside analog reverse-transcriptase inhibitor (NRTI), is a pivotal drug in the treatment of HIV/AIDS and chronic hepatitis B. Originally approved by the FDA in 1995 for HIV treatment, 3TC has since become a cornerstone in antiretroviral therapy (ART) regimens due to its potent antiviral activity, favorable safety profile, and synergistic effects when used in combination with other antiretrovirals [1]. Structurally similar to cytidine, 3TC interferes with viral DNA synthesis by causing premature chain termination, effectively inhibiting viral replication [2]. In HIV treatment, 3TC is often combined with other NRTIs such as tenofovir disoproxil fumarate, zidovudine, or abacavir, enhancing the efficacy and reducing the risk of resistance development. Its role in combination therapies is critical, given the high mutation rate of HIV and the consequent risk of resistance. Clinical trials have demonstrated significant improvements in viral suppression and CD4 cell counts in patients receiving 3TC-based regimens [3]. For chronic hepatitis B, 3TC provides substantial benefits by suppressing hepatitis B virus (HBV) DNA levels and improving liver histology. However, its long-term use is limited by the emergence of drug-resistant HBV strains, necessitating careful monitoring and, in some cases, combination with other antiviral agents [4]. 3TC’s efficacy and safety extend to pediatric use, making it a critical component in the treatment of HIV-infected children. Studies have shown that 3TC is well tolerated in children, with a safety profile comparable to that observed in adults [5,6]. The inclusion of 3TC in pediatric ART regimens has significantly improved outcomes, contributing to better viral suppression and immune recovery in this vulnerable population [7].

3TC is widely used alone or in combination with other anti-HIV drugs in infant to adolescent age groups of pediatric populations. It is a BCS class III drug with high solubility in water (70 mg/mL) [8,9]. It is also very stable in water under neutral pH with 82% bioavailability in adults and 68% bioavailability in pediatric patients [10,11]. It is commercially available as a solution (10 mg/mL), tablet (150/300 mg strengths), and fixed-dose combination with a number of anti-HIV drugs. Dose of the drug is calculated based on age and/or body weight of the child and varies from 4–10 mg/Kg for infants and to children ≤ 3 years and 75–150 mg/Kg for children weighing 14 Kg to ≤25 Kg when administered alone. The required dose can be easily administered to various groups using the available solution of 3TC. Fixed-dose combinations are available in a single or double-strength dose, which makes dosing difficult to adjust. For example, only one strength of tenofovir disoproxil fumarate and 3TC (300 mg/300 mg) is available in tablet form, making it difficult to adjust the dose. The usual practice is to use the available tablets to dispense as a liquid formulation. Even though 3TC is highly water soluble and stable, dispensing the correct amount of liquid formulation is still challenging due to the lower dose requirements for infants. Additionally, many issues have been reported for the compounded formulation, such as assay, stability, and bioavailability, due to a lack of quality control for compounded medications [12]. The advent of 3D printing technology offers innovative approaches to manufacture dose-flexible, quality formulations. The 3D printing of 3TC allows for the customization of dosage forms tailored to individual pediatric patients’ needs, addressing challenges such as dose flexibility and palatability. This technology enables the creation of tablets with precise doses, potentially improving adherence and therapeutic outcomes in pediatric populations [13]. Selective laser sintering is a laser- and heat-based technique that can be used to 3D print drugs with thermal resistance to degradation, such as 3TC. The objective of the current work is to demonstrate utility of printing dose-flexible formulations of 3TC that can be implemented to combination drugs.

## 2. Results and Discussion

### 2.1. Preliminary Results

The range of process parameters and the formulation composition were determined by the results of the initial trials. The selection of surface and chamber temperature ranges was based on the melting point and thermal stability of the 3TC. The melting point of 3TC is 179.2 °C [14,15,16,17,18] and it is thermally stable up to 232–315 °C [19]. Printing below 130 °C resulted in the edges of the printed layers warping and being pushed off by the roller. This was due to the thermal shock that the newly sintered layer experiences when the temperature difference between surface of the powder and the freshly sintered area is too high [20]. The 3TC loading of 20% (*w*/*w*) resulted in excellent powder spreadability and well-defined printlets at a targeted surface temperature range of 130–150 °C. The chamber temperature range of 105–120 °C was selected for similar reasons. Temperatures higher than 150 °C resulted in the melting and, consequently, solidification of the powder in the printing and reservoir chambers, preventing the powder from being evenly deposited on the printing surface. The laser scanning speed range of 250–350 mm/s was found to be the working limit. Any value outside of this range either resulted in very weak or burnt printlets due to the faster or slower laser scanning speed. As for the formulation, Hypromellose and Kollicoat^®^ were found to create printlets with favorable physical attributes (i.e., hardness and disintegration). In addition to Crospovidone, which was used as a disintegrant, sucrose was also added into the formulation to help mask bitter of the 3TC. Sucrose and Hypromellose both have higher densities than Kollicoat^®^. However, powder segregation was not observed during powder layering and printing. The formulations were preheated for 15 min at their target temperatures before printing to ensure that the powder bed reached its target temperature. This was especially important for formulations printed with higher surface and chamber temperatures. After printing, the printlets were left to cool inside of the printer for 30 min to reduce the warping effect resulting from thermal shock [21].

### 2.2. Characterization of Printlets

#### 2.2.1. Weight, Assay, Hardness, and Disintegration Time

The weight, diameters, and thicknesses of the 3TC printlets ranged between 33.8 and 47.0 mg, 5.7 and 6.2 mm, and 2.8 and 3.2 mm, respectively. The drug content of the 3TC printlets was close to the theoretical drug concentration of 20% LPV and ranged between 93.1 and 103.5%.

**Figure 1:**
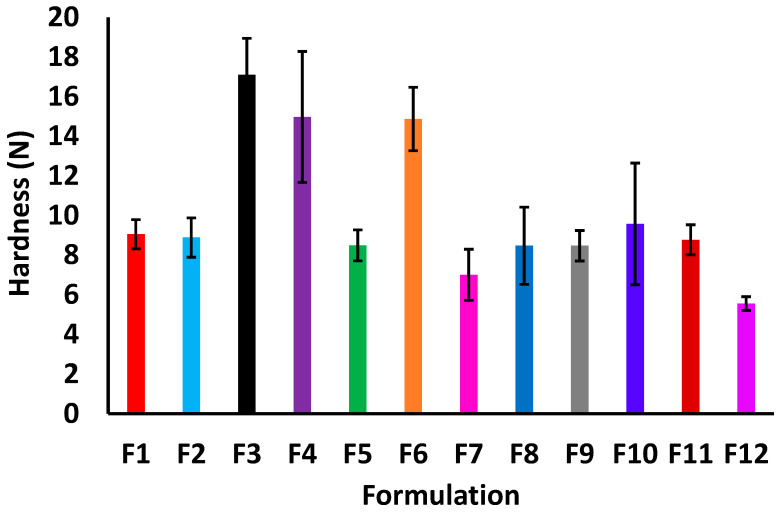
Hardness values of printlet formulations F1–12.

The hardness of the printlets varied from 5.6 ± 0.3 (F12) to 17.1 ± 1.8 (F3) N (Figure 1). None of the studied variables had statistically significant effects (*p* > 0.05) on the hardness. However, among the studied variables, laser scanning speed had prominent effect on the hardness. The formulations printed at slower scanning speeds were mechanically stronger than the printlets printed at faster printing speeds. The slow speed of printing resulted in a higher degree of sintering due to matrix softening and melting. This caused the matrix particles to fuse together to form a stronger and more consolidated structure. Additionally, sucrose and/or Hypromellose appeared to lower the hardness regardless of the laser scanning speed. For this reason, hardness seems to have no correlation with the dissolution of the printlets.

**Figure 2:**
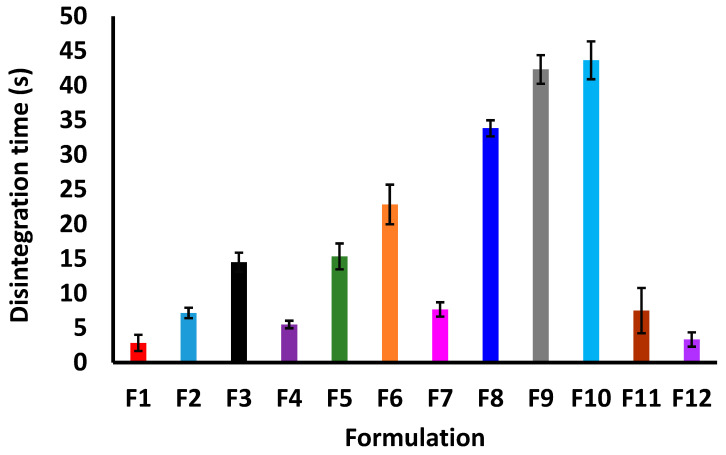
Disintegration time of printlet formulations F1–12.

The DT varied from 2.8 ± 1.2 (F1) to 43.7 ± 2.7 (F10) s (Figure 2). Low values of the disintegration times were due to the increased surface area resulting from the highly porous nature of the printlets printed by the process. Similar to hardness response, none of the studied variables had statistically significant effect on the DT (*p* > 0.05). However, DT was adversely affected by laser scanning speed and Hypromellose percentage in the formulations. A slower laser scanning speed decreased the hardness and, thus, prolonged disintegration. On the other hand, Hypromellose prolonged disintegration though a different mechanism. Hypromellose jellified in aqueous media and binds the matrix particles, thus hindering disintegration. Contrarily, the sucrose fastened disintegration due to its high solubility in water, thus acting as a disintegrant.

#### 2.2.2. Dissolution

**Figure 3:**
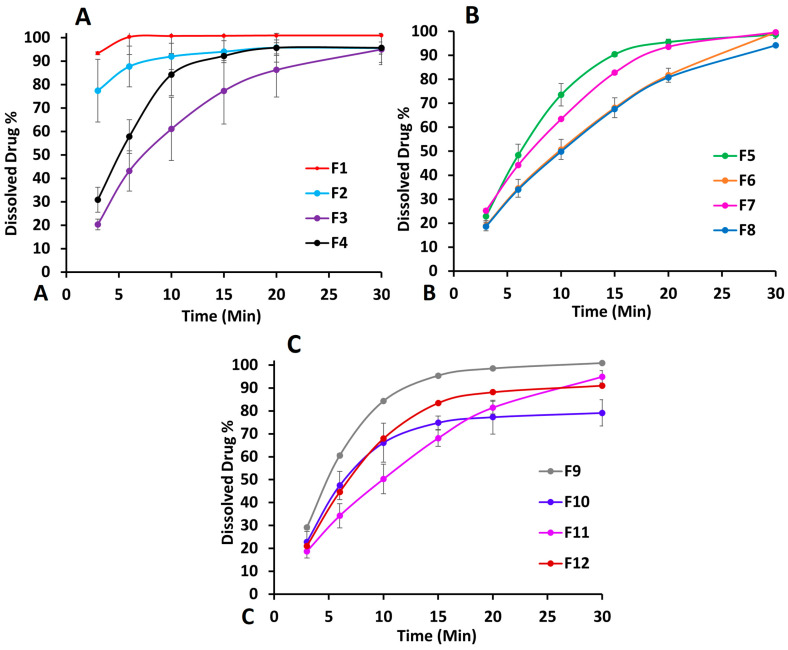
Dissolution vs. time profiles of printlets (**A**) F1–F4, (**B**) F5–F8, and (**C**) F9–F12.

3TC dissolution should be performed in water or 0.1 N HCl for 30 min as per the USP monograph, depending on the tablet strength [22]. Furthermore, the tablets should dissolve more than 85% in 30 min. The dissolution was more than 85% in 30 min for all the formulations except F10. It varied from 79.1 ± 5.7 (F10) to 100.9 ± 7.8% (F1) in 30 min (Figure 3). Among the studied variables, surface temperature, chamber temperature, and Hypromellose percentage had statistically significant effects on the dissolution in 10 min (*p* < 0.05). Increasing the surface temperature and chamber temperature during the printing process decreased the dissolution. Similarly, the dissolution increased with an increase in the laser scanning speed during the manufacturing of the printlets. This can be explained through the effect of these variables on the degree of fusion, sintering, and melting during the printing process. The temperature of the chamber and the surface temperature also provide the energy required for sintering and melting. Similarly, increasing the laser scanning speed decreased the melting and sintering. Thus, decreasing the energy density caused a decrease in the degree of fusion and consolidation [23,24,25]. However, the presence of excipients, mainly Hypromellose, seems to have a statistically significant effect (*p* < 0.05) on the dissolution. When the dissolution profiles of formulations F1 and F4 (Figure 3A) were compared, Hypromellose significantly slowed down the dissolution of the drug in the first 10 min, regardless of the identical printing parameters. The difference in dissolution between these formulations was further accentuated by the presence of sucrose and Crospovidone in F1. Both excipients are highly water soluble and can cause rapid disintegration. A compounded effect of laser scanning speed and Hypromellose on the drug dissolution can be observed when the dissolution profiles of F9 and F10 are compared (Figure 3C). F10 was printed at a slower laser scanning speed and contained more Hypromellose with no added sucrose or Crospovidone. This resulted in significantly decreased dissolution, with ~11.9% less being dissolved after 30 min compared to F9 (Figure 3C). Furthermore, the effect of surface temperature had a significant effect (*p* < 0.05) on the dissolution compared to Crospovidone. This behavior was observed when the dissolution profiles of F6 and F7 were compared (Figure 3B). Despite exhibiting similar dissolutions at the end of 30 min, the dissolution in 10 min was higher in the F7 compared to the F6 sample. The effect of Hypromellose on dissolution can be mitigated by reducing the surface temperature and adding Crospovidone, as seen in F10 and F11 (Figure 3C).

#### 2.2.3. Differential Scanning Calorimetry

**Figure 4:**
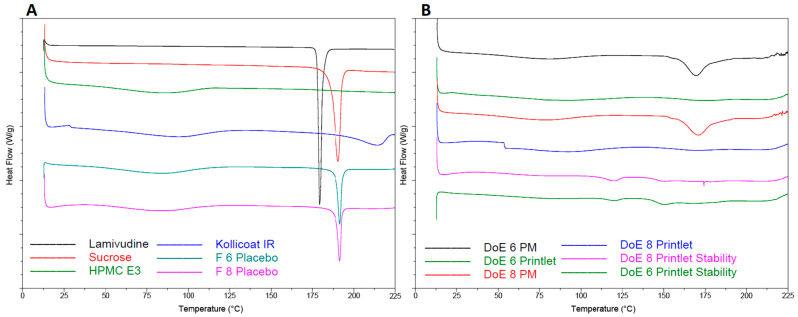
DSC thermograms of (**A**) lamivudine, excipients, and placebo F6 and F8 and (**B**) physical mixture (PM) and printlets F6 and F8 before and after exposure to stability conditions.

Among the excipients used in the printlet formulations, 3TC and sucrose were crystalline components as both exhibited melting endothermic peaks at 179.2 and 190.5 °C, respectively (Figure 4A). These values were similar to reported values in the literature [14,15,16,17]. On the other hand, Hypromellose and Kollicoat^®^ showed no endothermic peaks. Instead, broad, and shallow peaks were observed which are characteristics of amorphous or partially crystalline materials. Both showed shallow peaks below 100 °C, while Kollicoat^®^ also showed broad and shallow peaks at 212 °C [26]. The placebo formulations (F6 and F8) showed a melting peak at 191.5 °C, suggesting the presence of sucrose. On the other hand, PM (physical mixture) did not show melting peaks of the crystalline components at their respective melting points, but a broad melting peak was observed at 170 °C, which indicated a partially amorphous material (Figure 4B). However, this material could possibly be the drug as some of the drug might have dissolved in the softened polymer matrix upon heating. This partially amorphous matrix may also act as a solvent to dissolve sugar as no peak for sucrose was observed. The shifting of the drug peak from 179.2 to 170.9 °C was due to the presence of polymers that acted as impurities [27,28]. Unlike the placebo and PM, the printlet formulations did not show melting peaks for the drug or sucrose, indicating the amorphization of the drug and sugar during the printing process [29]. DSC thermograms of the formulations processed using different printing parameters or compositions did not show significant differences. This indicated that the components were completely amorphized or that the DSC technique was not sensitive enough to differentiate among the formulations processed differently.

#### 2.2.4. Fourier-Transform Infrared Spectroscopy

**Figure 5:**
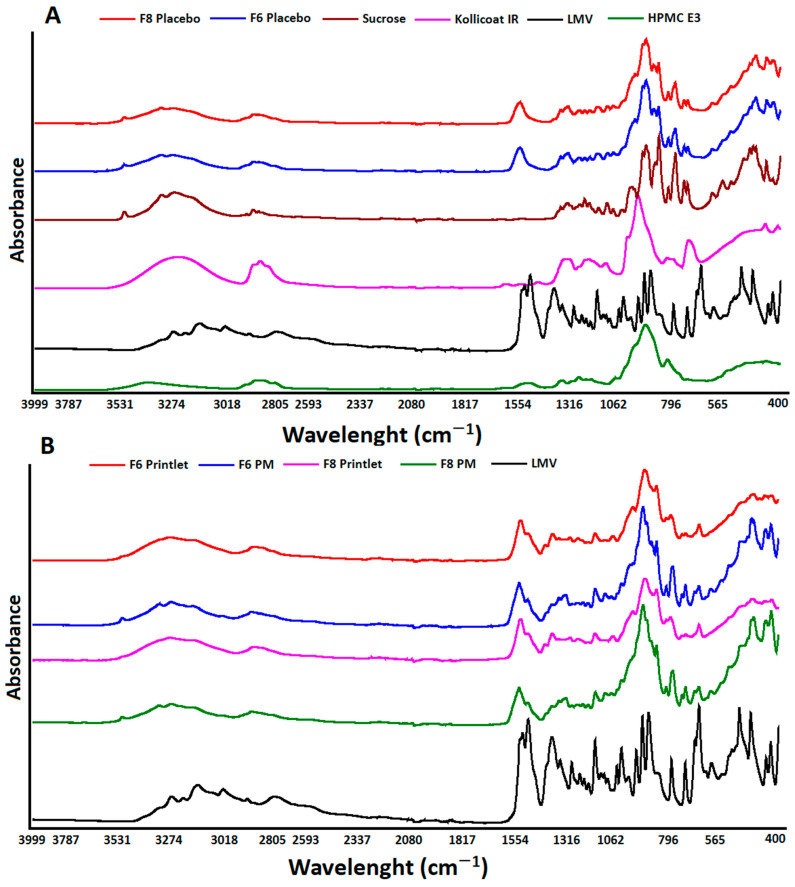
FTIR spectra of (**A**) lamivudine, excipients, and placebo F6 and F8 printlets, and (**B**) physical mixture and printlets F6 and F8.

The 3TC showed the characteristics stretching absorption bands due to C=O at 1646 cm^−1^ as a small notch, C-N at 1634 cm^−1^, C=C at 1606 cm^−1^, C-H at 1492 cm^−1^, amino group at 3325 cm^−1^, and hydroxyl at 3203 cm^−1^ (Figure 5A) [30]. The placebo showed additive spectra encompassing absorption bands of individual excipients used in the formulations. Similarly, the PM of the printlet formulation (powder before the printing process) showed additive spectra. However, not all absorption bands of the drug can be identified due to overlap of the bands between the drug and the formulation components. The drug absorption bands in the physical bands can be identified at 1610 and 786 cm^−1^, and bands at 1646 and 1634 merged to appear at 1650 cm^−1^ due to excipient absorbance band interference at 1657 cm^−1^ [31]. In the printlets, the absorption bands attributed to the drug and excipients broadened, reduced in intensity, or shifted to a new wave number. This indicated phase transformation of the 3TC (Figure 5B). These data concurred with the powder X-ray diffraction data.

#### 2.2.5. X-Ray Powder Diffraction

**Figure 6:**
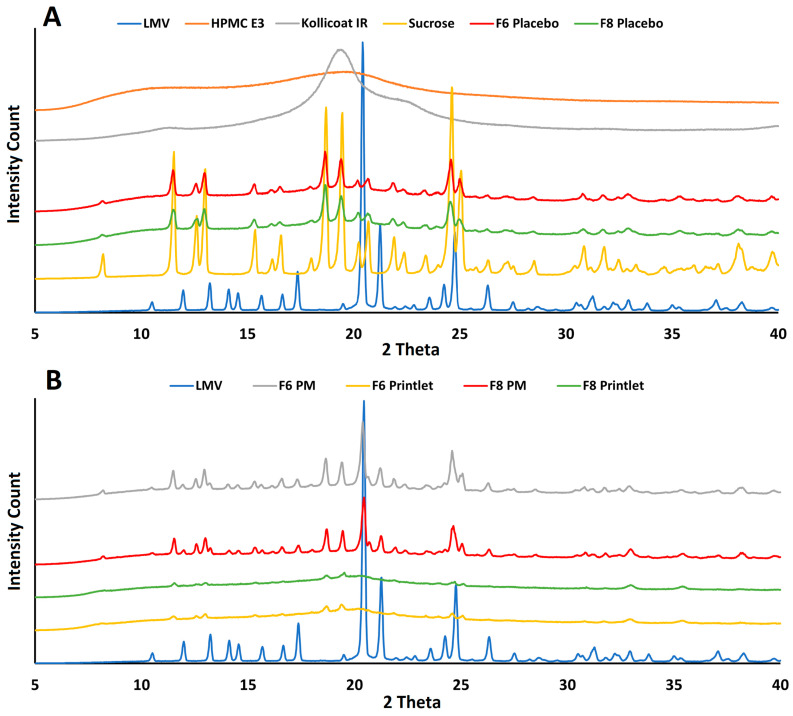
XRD diffractograms of (**A**) lamivudine, excipients, and placebo F6 and F8 and (**B**) physical mixture and printlets F6 and F8.

The 3TC exhibited major reflection peaks at 2θ value at 10.4, 12.0, 13.2, 14.1, 14.5, 15.7, 16.6, 17.3, 20.4, 21.2, 24.2, 24.7, and 26.3° and many minor peaks indicating its crystalline nature [32]. Sucrose showed multiple reflection peaks, while Hypromellose and Kollicoat^®^ showed halo diffractograms of a typical amorphous material (Figure 6A). The physical mixture of excipients (placebo) exhibited reflection peaks of sucrose as the other excipients were amorphous in nature. The powder formulations of the printlets before printing (PM) showed additive peaks of sucrose and 3TC (Figure 6B). Furthermore, many of the drug reflection peaks interfered with sucrose. Discrete peaks of the drug in the PM were identified at 10.4, 12.0, 13.2, 14.1, 14.5, 15.7, 17.3, 20.4, and 21.2°. The printlets exhibited distinguished low-intensity peaks of the drug, similar to the peaks observed in the physical mixture. However, the intensity of the reflection peaks reduced significantly and most of the peaks attributed to the drug disappeared for the printlets. Similarly, the reflection peaks of the sucrose significantly reduced or/and disappeared compared to those for the preprinted powder (PM). This indicated the partial amorphization of the drug and sucrose during the printing process [33]. The reduction in peak intensity was related to the studied process variables. Among all of the studied variables, laser scanning speed seemed to have a significant impact on the reflection peak intensity. The printlet formulations F6 and F8 were printed at laser scanning speeds of 350 and 250, respectively. The reduction in the peak intensity was higher for F8 compared to F6 due to the slower laser scanning speed used in F8 manufacturing. The slower scanning speed resulted in higher energy density over the scanned area of the powder bed and, consequently, a higher degree of sintering, melting, and transformation of crystalline material into the amorphous phase.

#### 2.2.6. Near-Infrared Hyperspectral Imaging

**Figure 7:**
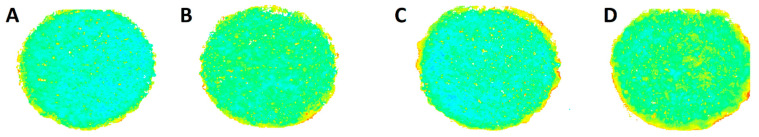
NIR chemical imaging pictures of printlet F6, top (**A**) and bottom (**B**); and printlet F8, front (**C**) and back (**D**).

Principal component analysis (PCA) concentration images of the printlets were generated by the baseline correction, mean centering, and standard normal variate analysis of hypercube data (Figure 7). The pixel colors represent the components of the formulation. Red and blue pixels mean high and low concentrations of a particular component, respectively. PCA is used for the qualitative analysis of component distribution in a matrix. The printlets showed uniform green-yellowish pixels at the top and bottom of the printlets, which indicated the uniform distribution of the components.

#### 2.2.7. Effect of Process Variables on Powder Properties

**Figure 8:**
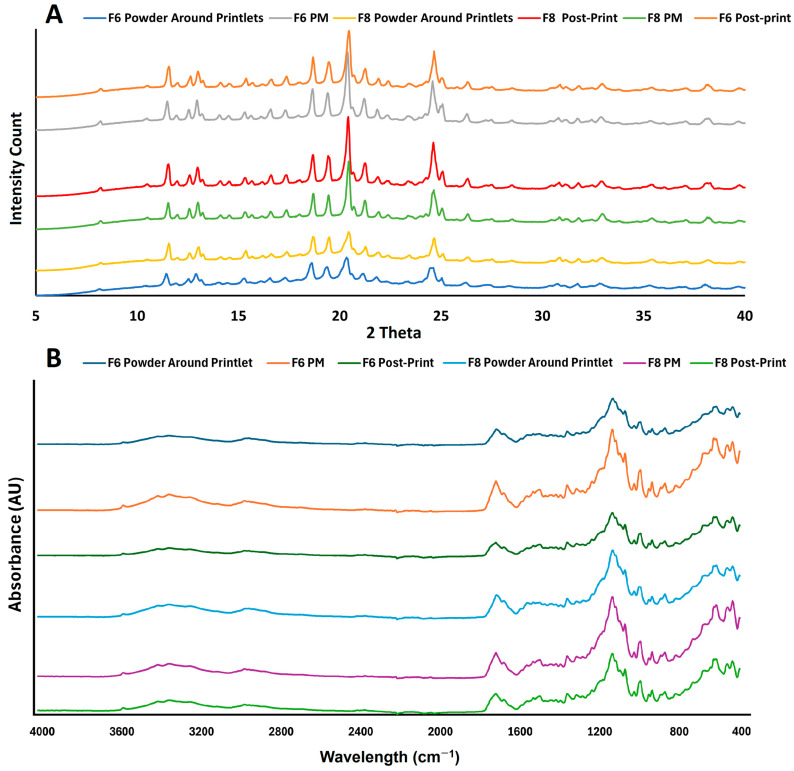
(**A**) X-ray powder diffractograms and (**B**) FTIR spectra of solidified powder around the printlets and pre-print (PM) and post-print powders for F6 and F8.

To understand the effect of the process variables on the unprinted powder that acted as a support material for the printlets, the powder material after the printing process was analyzed to measure the drug phase. The XRPD patterns of the powder formulations (Figure 8A) were similar before and after the printing process. However, a significant decrease in signal intensity was observed in the post-printed powder (the powder collected after the printing process) for both the drug and the sucrose. This indicated a decrease in crystallinity of the formulation components. Furthermore, the degree of decrease in the reflection peaks was also dependent on the printing process variables employed during printing. The most important variable affecting the reflection peak intensity was laser scanning speed. This was exemplified by the formulation powders F6 and F8. The decreases in the reflection peak intensity were 18.0 and 20.8% for F6 and F8, respectively, compared to the preprinted powder. Due to the exposure to high surface temperatures during printing, the formulations printed at 150 °C resulted in the solidification of the unsintered powder around the printlets. In some formulations (F6 and F8), loose powder was solidified enough to be lifted as a slab, encapsulating the printlets in it. When the printlets were collected, the unsintered solidified powder around the printlets was collected and further analyzed. This powder showed a higher decrease in reflection peaks compared to the pre-and post-printed powders. The decreases in intensity of the powder collected around the printlets from F6 and F8 were 67.2 and 68.1%, respectively. This indicated that the powder exposed to the printing process had a reduced degree of crystallinity. This was due to the combination of the surface temperature and laser, which may have caused the deformation of the crystalline materials. The FTIR spectra of the pre- and post-printed powders appeared similar, indicating no significant change (Figure 8B). However, the spectra of the powder around the printlets showed similar spectra of the printlets themselves, indicating the partial amorphization of the drug and excipients of the formulations.

#### 2.2.8. Stability

**Figure 9:**
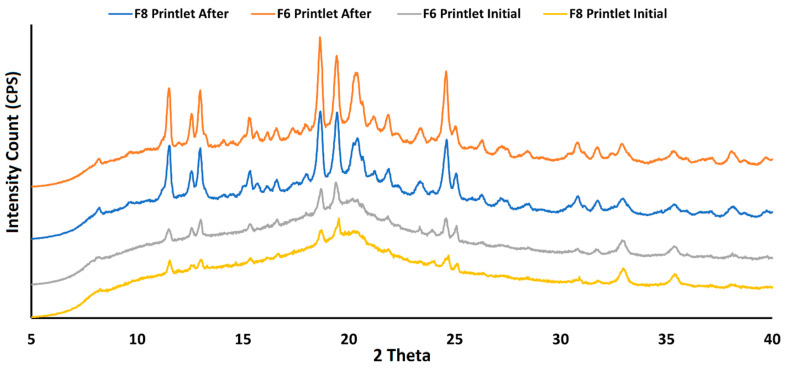
XRPD diffractograms for initial and stability samples of printlets F6 and F8.

The DSC of the printlets exposed to stability conditions did not show significant differences compared to the initial samples (Figure 4B). However, the samples showed low-intensity broad and shallow humps at about 125 and 150 °C that may be related to the amorphous matrix. The FTIR results did not show significant changes in the spectra; however, the XRPD diffractogram of the stability samples showed an increase in refraction peak intensity compared to the initial printlets (Figure 9). This indicated the crystallization of amorphous material during the stability period.

**Figure 10:**
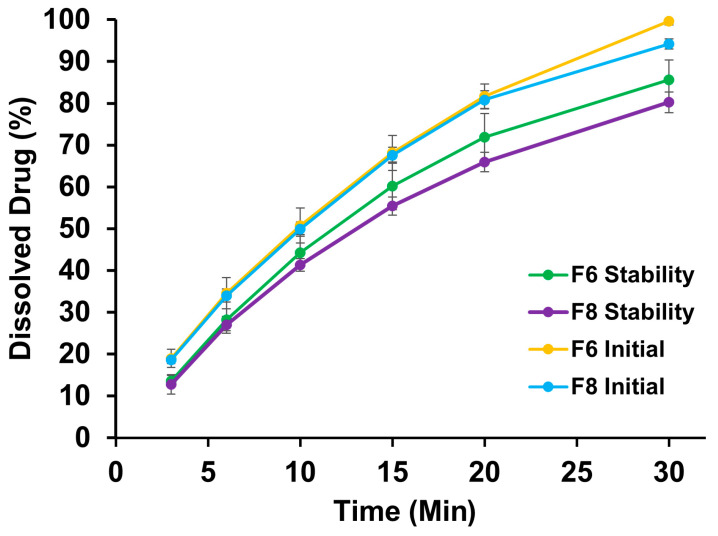
Dissolution vs. time profiles for initial and stability samples of printlets F6 and F8.

There was a slight decrease in assay value after a year of storage at room temperature. The assay of the F6 and F8 printlets decreased from 95.9 ± 1.9 to 95.1 ± 1.2 and 97.4 ± 3.8% to 93.9 ± 1.0, respectively. The DT of the F6 and F8 printlets increased from 22.8 ± 2.9 to 67.8 ± 9.2 s and 33.8 ± 1.2 to 152 ± 22.7 s, respectively. Similar to the assay value, the dissolution of the F6 and F8 printlets decreased from 99.6 ± 0.9 to 85.6% 94.2 ± 1.2 to 80.2 ± 2.5%, respectively (Figure 10). The prolongation of DT and decrease in dissolution can be explained by moisture being absorbed into the printlet matrix of the Hypromellose polymer, resulting in greater bonding among the components of the printlets.

#### 2.2.9. Pharmacokinetics

3TC undergoes multiple intracellular transformations to achieve a therapeutically active form. The intracellular activation process consists of two main steps: cellular uptake and phosphorylation cascade. The cellular uptake of 3TC is mainly facilitated by passive diffusion, although some studies showed the involvement of transporters [34,35]. After cellular uptake, a three-step phosphorylation cascade converts 3TC from its base form to an active triphosphate form. 3TC converts to mono-, di- and triphosphate form by the cellular kinases deoxycytidine kinase, nucleotide kinase, and nucleoside diphosphate kinase, respectively [36,37].

**Figure 11:**
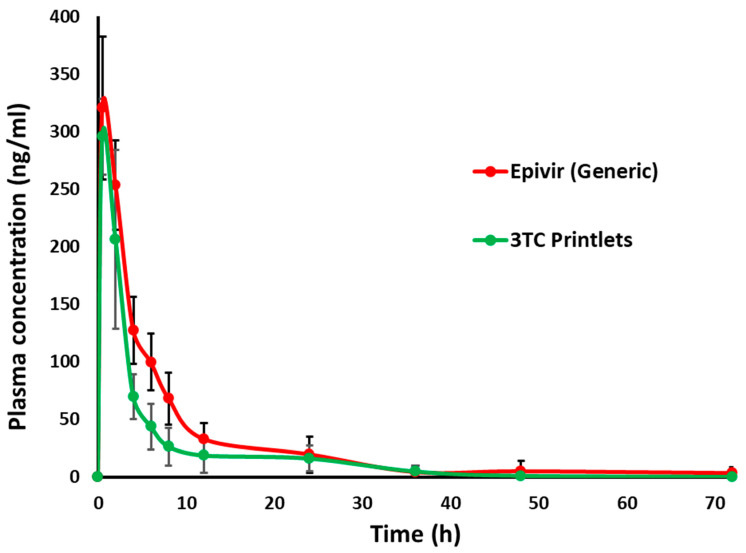
Comparative pharmacokinetic profiles of 3TC-loaded printlet (F7) and FDA-approved generic tablets.

The plasma concentration profiles of the 3D printed 3TC printlets F7 (test product, T) and commercial tablets (reference product, R) were similar to each other as the curves superimpose each other (Figure 11). Furthermore, no statistical significance (*p* < 0.05) was observed between pharmacokinetic parameters such as maximum plasma concentration (C_max_), time to reach maximum plasma concentration (T_max_) and area under the curve (AUC). The C_max_, T_max_, and AUC of the printlets and commercial tablets were 295.5 ± 33.0 and 305.0 ± 70.1 ng/mL, 0.5 ± 0.0 and 1.0 ± 0.8 h, and 1414.1 ± 174.0 and 1987.2 ± 700.5 ng.h/mL, respectively. Both formulations maintained plasma concentrations of more than 25 ng/mL for up to 12 h. The median EC_50_ values of 3TC against HIV-1 varied from 20–60 nM. Both formulations achieved plasma concentrations of the drug of more than 60 nM at 0.5 h. Minor differences in pharmacokinetic parameters were observed between genders. However, the differences were statistically insignificant (*p* > 0.05). For example, higher values of T_max_, C_max_, and AUC were observed in male rats compared to female rats. This could be related to kidney function, as 3TC is eliminated unchanged through the kidneys [34].

A bioequivalence comparison was conducted between the two formulations, as per FDA guidance documents [12]. C_max_ and AUC were log-transformed and used to calculate the geometric mean and 90% confidence intervals (CI). The 90% CI of C_max_ and AUC were 0.973–1.032 and 0.961–1.043, respectively. The T/R and R/T ratio of the geometric mean of the C_max_ and AUC were 0.998 and 1.002, and 0.960 and 1.041, respectively. Thus, the values of the R/T and T/R ratios of the C_max_ and AUC fell within the 90% CI. Two formulations can be considered bioequivalent when the CI of the T/R or R/T ratio of C_max_ and AUC falls between 0.8 and 1.25, according to FDA guidance. Therefore, the two formulations can be considered bioequivalent as they met the FDA bioequivalence criteria [12].

## 3. Materials and Methods

### 3.1. Materials

3TC (AK scientific, Union City, CA, USA), Candurin^®^ NXT Ruby Red (Merck, Darmstadt, Germany), hydroxypropyl methylcellulose E3 (HPMC, Hypromellose) (JRS Pharma, Rosenberg, Germany), sucrose (Acros Organics, Geel, Belgium), Kollicoat^®^ IR (ethylene glycol and vinyl alcohol graft copolymer), and Kollidon^®^ CL-M (Crospovidone) (BASF Chemicals company, Ludwigshafen, Germany) were used. Ammonium acetate, acetonitrile, methanol, sodium edetate, and formic acid were obtained from Fisher Chemical Hampton, NH. Glacial acidic acid was purchased from VWR Analytical, West Chester, PA. All reagents were of analytical grade and used as received. In-house 18 MW water (deionized water) obtained from the Milli-Q Gradient A-10 water purification system (Millipore Corporation, Bedford, MA, USA) was used in all studies.

### 3.2. Methods

#### 3.2.1. Printing of Printlets

The range of process parameters and selection of excipients was based on the preliminary experiments and previous published work [13,38,39]. The effect of formulation and process parameters on the quality of the printlets was studied by the design of the experiment approach. Based on the preliminary data, surface temperature, chamber temperature, laser scanning speed, sucrose, polymer, and disintegrant concentrations were selected as independent variables. An experiment design matrix was created using the Plackett–Burman fractional factorial design of experiment with twelve experiments. The upper and lower limits for all independent variables are listed in Table 1. Laser scanning speed (X_1_), surface temperature (X_2_), chamber temperature (X_3_), sucrose (X_4_), Hypromellose (X_5_) and Crospovidone (X_6_) concentrations of 150–130 °C, 120–105 °C, 250–350 mm/s, 0–30%, 0–42%, and 0–5% were selected for the study, respectively. A powder mixture of 120 g of each formulation was prepared and all formulations contained 20% 3TC and 3% Candurin^®^ NXT Ruby Red. Kollicoat^®^ was used as a filler (Table 2). All components were sieved through a #100 ASTM sieve and mixed for 5 min in a plastic bag. The powder was loaded into reservoir chamber of an SLS printer (Sintratec Kit, AG, Brugg, Switzerland) equipped with a 2.3 W blue diode laser (445 nm). A 3D model of the printlets with a height of 3 mm and diameter of 6 mm was modeled using Solidworks (Solidworks Inc., Waltham, MA, USA. The printlets were printed as per the experiment matrix while keeping the layer thickness constant (0.15 mm) [38,39]. The weight, diameter, and thickness of the printlets were evaluated using a digital caliper.

#### 3.2.2. Disintegration Time

United States Pharmacopeia (USP) disintegration apparatus (Vankel Varian VK-100, NC) was used to determine the disintegration time (DT) of the printlets. The printlets (*n* = 6) were placed in the basket and submerged in water at 37 ± 1 °C. The time required to complete the disintegration of the printlets was recorded in seconds.

#### 3.2.3. Dissolution

USP-II apparatus (Model 708-DS with 850-DS autosampler, Agilent Technologies, Santa Clara, CA, USA) equipped with a mini dissolution vessel (200 mL) and mini paddle was used to perform the dissolution of the printlets (*n* = 3). The printlets were placed in 200 mL of 0.1 N HCl solution. The paddle speed was fixed at 50 rpm and dissolution tests were conducted at 37 ± 0.5 °C. A volume of 1 mL sample was collected and replaced with equal amounts of fresh media at 3, 6, 10, 15, 20, and 30 min. The percentage of 3TC dissolved from the printlets was determined by the validated HPLC method.

#### 3.2.4. Differential Scanning Calorimetry

A Q2000 instrument (TA Instruments Co., New Castle, DE, USA) was used to collect differential scanning calorimetry (DSC) data. Approximately 4 mg samples of 3TC, Hypromellose, Kollicoat^®^, sucrose, physical mixture (PM), placebo, and the printlets were weighed into aluminum pans and crimp-sealed before being loaded into the equipment. The temperature scanning rate was 10 °C/min and the samples were scanned up to 225 °C for DSC measurement. An inert atmosphere during the measurement was provided via nitrogen gas that was purged at a pressure of 20 psi and a 20 mL/min flow rate. The DSC equipment was calibrated using Indium with a transition temperature of 156.6 °C to ensure the accuracy of the measurement.

#### 3.2.5. Fourier-Transform Infrared Spectroscopy

A Nicolet^TM^ IS^TM^ 50 system (Thermo Fisher Scientific, Austin, TX, USA) was used to collect Fourier-transform infrared (FTIR) spectra of the samples. The data collection parameters were absorbance mode, a wavenumber range of 400–4000 cm^−1^, a data resolution of 8 cm^−1^, and 32 scans. OMNIC software version 9.0 (Thermo Fisher Scientific, Austin, TX, USA) was used to capture and analyze the spectra.

#### 3.2.6. X-Ray Powder Diffraction

A Bruker D2 Phaser SSD 160 Diffractometer (Bruker AXS, Madison, WI, USA) was used to collect the X-ray powder diffraction (XRPD) patterns of the samples. A 400 mg powder sample was evenly filled and packed into the sample holder to prevent crystal preferential orientation. The 2θ range of 5–40° was scanned for the samples with a step size of 0.0202 at 2 s per step (3000 steps in total). The rotation speed of the samples was 15 rpm/min to obtain an average diffractogram of the sample. The data were analyzed using Diffrac.EVA Suite version V4.2.1 (Bruker AXS, Madison, WI, USA).

#### 3.2.7. Near-Infrared Hyperspectral Imaging

A Via-Spec SWIR control system was used to control the cryogenically cooled mercury–cadmium–telluride sensor spectral camera via a camera link interface. The camera captures images at a resolution of 384 × 288 pixels, with each pixel measuring 24 × 24 μm. The printlets were positioned on a metal sample holder and placed on a movable stage, which was illuminated by a halogen line light source from Via-Spec II. The instrument employs a push-broom configuration to generate a chemical image. As the stage moves upward beneath an imaging spectrograph, it captures simultaneous spectral measurements from a sequence of adjacent spatial positions. The distance between the lens and the object was carefully adjusted to ensure proper focus on the sample, optimizing image quality in reflectance mode. Data were collected with an integration time of 7.005 ms, a frame rate of 75 MHz, and a scan axis speed of 0.2 inches per second. Prior to sample measurement, reference and background images consisting of pure white and dark images were obtained. Middleton Spectral Vision was used for data acquisition, while data analysis was performed using Prediktera Evince™ (Prediktera AB, Umea, Sweden).

#### 3.2.8. Stability

The stability of the printlets was determined by storing the printlets at room temperature in pharmacy vials at approximately 22–23 °C/45–50% relative humidity for a year. The printlets were characterized for physicochemical attributes.

#### 3.2.9. Bioequivalence Study

The pharmacokinetic study was conducted using commercial 3TC tablets (reference) and the printlet formulation (test). Sprague Dawley rats (*n* = 6/group, male/female 1:1) weighing 250–350 g was used as subjects for the study. The rats were subjected to food and water fasting during the study. The study was performed as per the protocol #IACUC 2021–0215 approved by Texas A&M’s Institutional Animal Care and Use Committees. A suspension of the printlets and tablet formulations were administered to the animals at 30 mg/Kg by oral gavage. The tail vein was used to collect approximately 0.3 mL blood, which was stored in a sodium-edetate-coated centrifuge tube. Blood samples were collected at 0, 0.5, 2, 4, 6, 8, 12, 24, 36, 48, and 72 h. The animals were subjected to isoflurane inhalation in order to anesthetize them before sample collection. By centrifuging the blood at 13,300 rpm and 4 °C for 15 min, plasma was separated. The plasma was stored at −80 °C before analysis.

3TC was extracted from the plasma sample using the protein precipitation method. Briefly, 125 μL of methanol was added to the 50 μL plasma followed by the addition of 10 μL of water and 15 μL of 0.01% formic acid solution, vortexed for 1 min, and centrifuged at 13,300 rpm and 4 °C for 20 min. Then, 100 μL of supernatant was transferred to a 3 mL centrifuge tube and dried completely under a steady flow of 5 psi nitrogen gas in a 50 °C water bath for 20 min. After drying, the samples were reconstituted in 100 μL of 97:3 0.01% formic acid/methanol by vortexing for 1 min. The reconstituted samples were then transferred into 300 μL UPLC vials and were analyzed using the validated UPLC-MS method.

#### 3.2.10. HPLC Method

The HPLC system consisted of an Agilent 1260 Infinity II (Agilent Technologies, Wilmington, DE, USA) equipped with a quaternary pump, online degasser, column heater, autosampler, and diode array detector (DAD). The elution was isocratic with a mobile-phase composition of methanol and 24.6 mM ammonium acetate buffer solution (pH 3.8; 5:95 *v*/*v*). The column temperature was maintained at 30 °C. Separation was achieved on a Luna CN (100 × 4.6 mm^2^), 3 µm particle size column (Phenomenex, Torrance, CA, USA). A sample volume of 20 µL was injected, and the analyte was detected at 270 nm. The data collection and analysis were performed using OpenLAB Data Analysis build 2.199 (Agilent Technologies, Wilmington, DE, USA).

#### 3.2.11. Ultra-Performance Liquid Chromatography–Mass Spectrometry

The 3TC extracted from the plasma samples was separated on an Aquity UPLC BEH phenyl (2.1 × 100 mm, 1.7 μm) (Phenomenex, Torrance, CA, USA) column using the Acquity^®^ UPLC system (Waters Corporation, Milford, MA, USA). The UPLC-MS was equipped with a quaternary pump, PDA, and QDa detectors. The separation of the samples was performed using a gradient method with an eluting mixture of 0.01% formic acid and methanol (97:3 *v*/*v*) for the first 4 min. From 4 min until 6 min, the eluting solution was switched to formic acid and methanol (3:97 *v*/*v*). From 6 min to 15 min, the eluting solution was again switched to the initial conditions of formic acid and methanol (97:3 *v*/*v*). The column temperature was maintained at 40 °C with the mobile phase flowing at 0.3 mL/min. The 3TC was eluted in 2.1 min. The parameters used for the mass spectrometry were electrospray positive ionization (ESI+) mode with 0.8 KV capillary voltage and 15 V collision energy. The mass of the 3TC was monitored using a QDa detector at 230.26. The calibration range of the 3TC was 25–1000 ng/mL.

#### 3.2.12. Statistical Analysis

Data were presented as mean ± standard deviation. Statistical significance was determined using the *p*-value. *p* < 0.05 was deemed statistically significant, while *p* > 0.05 was deemed statistically insignificant.

## 4. Conclusions

The SLS-printed lamivudine printlets disintegrated quickly and met USP assay and dissolution criteria. The addition of sucrose did not have any significant effect on the performance of the printlets other than a slight boost to the disintegration time. Interestingly, it was observed that the high surface temperatures during printing caused the crystallinity of the unsintered powder to change. The printlets were stable when exposed to room-temperature conditions for a year. The disintegration time of the printlets and, consequently, the dissolution values were slightly lower after 30 min after stability exposure. However, pharmacies can dispense the medication for up to 90 days and the drugs are expected to meet dissolution specifications during the in-use period. Moreover, the pharmacokinetic profiles and parameters (C_max_, AUC, t_1/2_) of the printlets and FDA-approved formulations were similar and met the bioequivalence criteria of 90% CI for the C_max_ and AUC. Thus, selective laser sintering can be employed to print quality printlets of lamivudine intended for use in pediatric patients. This method can also be applied in the printing of other thermally stable drugs intended for pediatric patients.

## Figures and Tables

**Table 1:** Upper and lower limit of independent variables.

Parameter Range	Laser Scanning Speed (mm/s)	Surface Temperature (°C)	Chamber Temperature (°C)	Sucrose (%*w*/*w*)	HPMC (%*w*/*w*)	Kollidon^®^ (%*w*/*w*)
Low	250	130	105	0	0	0
High	350	150	120	30	42	5

**Table 2:** Experimental design matrix and composition of printlet formulations.

Formulation	Laser Scanning Speed (mm/s)	Surface Temperature (°C)	Chamber Temperature (°C)	Sucrose (%*w*/*w*)	HPMC (%*w*/*w*)	Kollidon^®^ (%*w*/*w*)	Lamivudine (%*w*/*w*)	Candurin^®^ (%*w*/*w*)	Kollicoat^®^ (%*w*/*w*)
F1	350	130	105	30	0	5	20	3	42
F2	250	130	105	30	0	0	20	3	47
F3	350	150	120	0	0	0	20	3	77
F4	350	130	105	0	42	0	20	3	35
F5	250	130	120	0	0	5	20	3	72
F6	350	150	120	30	42	5	20	3	0
F7	350	130	120	30	42	0	20	3	5
F8	250	150	105	30	42	5	20	3	0
F9	350	150	105	0	0	5	20	3	72
F10	250	150	105	0	42	0	20	3	35
F11	250	130	120	0	42	5	20	3	30
F12	250	150	120	30	0	0	20	3	47

## Data Availability

The data presented in this study are available on request from the corresponding author.
